# Performance comparison of heuristic algorithms for task scheduling in IaaS cloud computing environment

**DOI:** 10.1371/journal.pone.0176321

**Published:** 2017-05-03

**Authors:** Syed Hamid Hussain Madni, Muhammad Shafie Abd Latiff, Mohammed Abdullahi, Shafi’i Muhammad Abdulhamid, Mohammed Joda Usman

**Affiliations:** 1 Faculty of Computing, Universiti Teknologi Malaysia, Skudai, Johor, Malaysia; 2 Department of Computer Science, Federal Urdu University Arts, Science and Technology, G-7/1, Islamabad, Pakistan; 3 Department of Mathematics, Ahmadu Bello University,Zaria, Kaduna State, Nigeria; 4 Department of Cyber Security Science, Federal University of Technology, Minna, Niger State, Nigeria; 5 Department of Mathematics, Bauchi State University, Gadau, Nigeria; University of Texas at San Antonio, UNITED STATES

## Abstract

Cloud computing infrastructure is suitable for meeting computational needs of large task sizes. Optimal scheduling of tasks in cloud computing environment has been proved to be an NP-complete problem, hence the need for the application of heuristic methods. Several heuristic algorithms have been developed and used in addressing this problem, but choosing the appropriate algorithm for solving task assignment problem of a particular nature is difficult since the methods are developed under different assumptions. Therefore, six rule based heuristic algorithms are implemented and used to schedule autonomous tasks in homogeneous and heterogeneous environments with the aim of comparing their performance in terms of cost, degree of imbalance, makespan and throughput. First Come First Serve (FCFS), Minimum Completion Time (MCT), Minimum Execution Time (MET), Max-min, Min-min and Sufferage are the heuristic algorithms considered for the performance comparison and analysis of task scheduling in cloud computing.

## Introduction

Cloud computing has become one of the most attractive fields in both ICT (Information and Communication Technology) trade and academic research. Some of the functions and services of cloud computing environment include advanced security, geographical distribution of large scale data, resilient computing, virtualization, web infrastructure, Web 2.0 and other developing technologies. With cloud computing technology, users can access provision, process, store and network important computer resources, operating systems, virtual desktops, web services, development platforms and databases. It also uses specific applications as services offered by cloud computing providers as a “utility” on “pay as you go”. Many benefits of the cloud computing environment include cost saving, energy efficiency, flexibility, high accessibility, rapid implementation and scalability [1–3].

Within the domain of computing, there are various kinds standard practices being followed based on inventions and technological advancement. The computing have various paradigm including the High Performance Computing (HPC), Parallel Computing, Distributed Computing, Cluster Computing, Grid Computing, Cloud Computing, Mobile Computing, Quantum Computing, Fog Computing, Bio Computing, Optical Computing, Nano Computing. As computing systems become more capable and faster, it requires the feature of modern computing, optimum scheduling and highly security [4–7].

**Fig 1:**
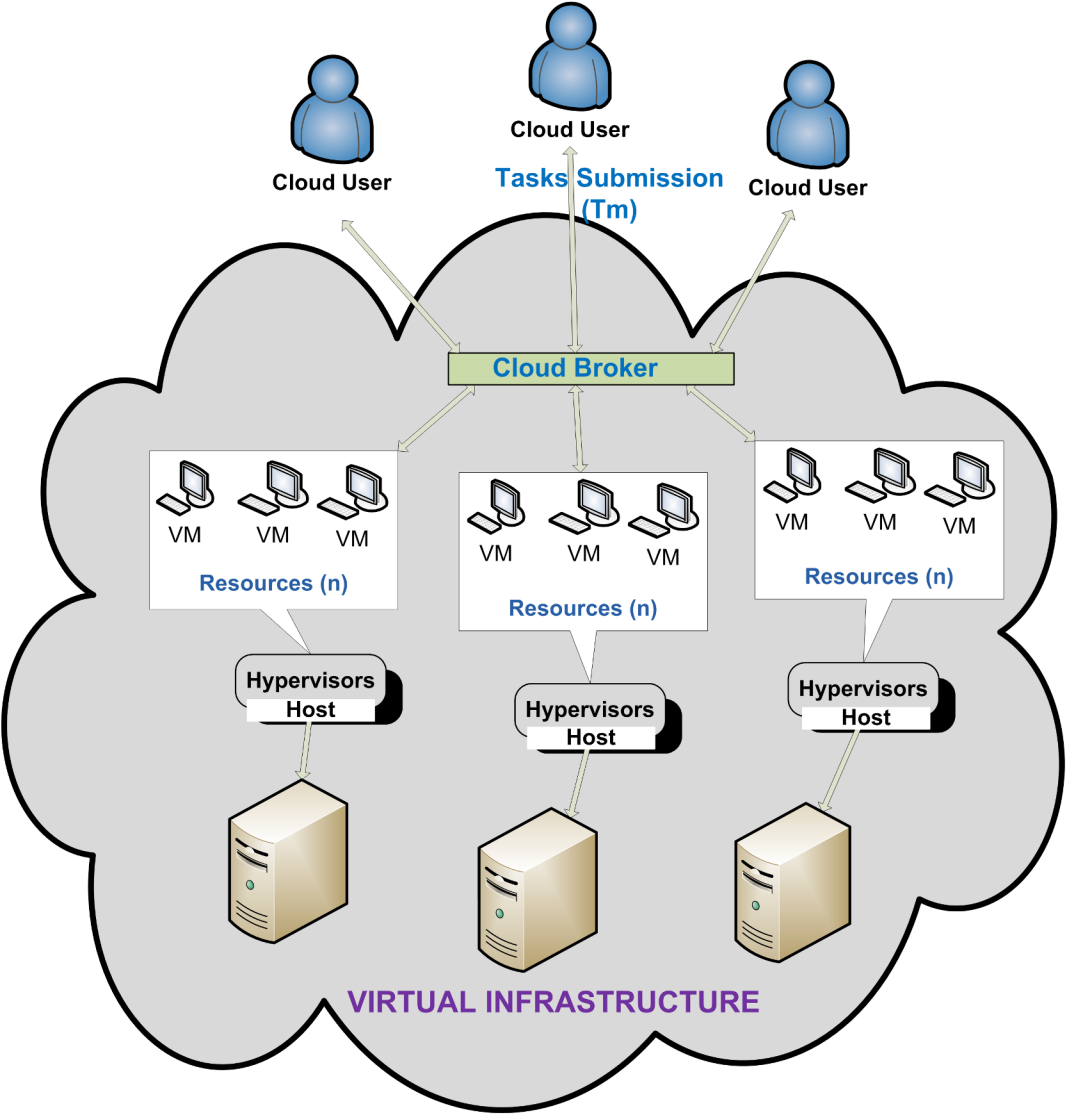
Task scheduling in IaaS cloud computing.

Task scheduling algorithms have a direct effect on the proficiency of users’ tasks and also in efficient utilization of resources in IaaS cloud computing environment. Hence, how to realize the optimum distribution of users’ tasks is still an unresolved question for task scheduling in this environment, as shown in Fig 1. The algorithm of task scheduling as implemented in the field of cloud computing is as follows: Initially, resources and tasks are mapped regarding to the existing task and information of resources in accordance with basic approaches or methods. At that point, tasks are mapped among the quality of service requirements of cloud users and the resources are distributed to the application of the task to confirm the competence of the task. In conclusion, the summary of the consequences is implemented by submitting the users’ demand [8, 9].

Optimal task scheduling in cloud computing environment is known to be an NP-complete problem [10, 11]. Existing heuristic algorithms for tasks scheduling are difficult to compare due to the contrasting underlying assumption by each heuristic algorithm. In this paper, we selected six rule-based heuristics from literature to consist of First Come First Serve (FCFS), Minimum Completion Time (MCT), Minimum Execution Time (MET), Max-min, Min-min and Sufferage. These heuristic algorithms are implemented in both homogeneous and heterogeneous environment with the help of CloudSim toolkit [12]. The results of the simulation for all the heuristics are considered under the same assumption.

Independent tasks are used for scheduling which is done off-line, that is the execution times of the tasks are known a priori. The metrics of performance comparison considered are cost, degree of imbalance, makespan and throughput. Tasks are executed on Virtual Machines (VM) in order of their arrival time and only one task is implemented on a VM at a time and pre-emption is not allowed. The number of tasks and VMs are known beforehand. This paper intends to provide a basis for evaluation and insights into situations, where one scheduling heuristic will implement better than the other.

The main objective of this paper is to explore heuristics algorithms for task scheduling and draw a contrast among them so as to arrive at a conclusion about the best available heuristic algorithm for cloud environment. The remaining sections of this paper are systematically organized as follows: In next section, we reviewed studies of task scheduling in the area of IaaS cloud computing. We chronicle the description of rule-based scheduling heuristic algorithms in the methodology section. Results and discussion show performance evaluation of heuristic algorithms with the help of experimental simulation. The last section consists of details the conclusion, recommendation and future works.

## Related works

In this section, we reviewed current studies which use the different heuristic, meta-heuristic and hybrid algorithms [13] for task scheduling in IaaS cloud computing system. Abdullahi, et al. [14] present a Discrete Symbiotic Organism Search (DSOS) algorithm for an ideal schedule of tasks on resources in cloud computing system. Experimental outcomes reveal that the DSOS performs better than Particle Swarm Optimization (PSO) in term of convergence rate. Furthermore, Abdullahi and Ngadi [15] present a hybrid Simulated Annealing (SA) and Symbiotic Organisms Search (SOS) algorithm called SASOS to attain optimum scheduling of tasks in cloud computing. The result proves that the suggested algorithm outperformed DSOS to achieve better convergence ratio and quality of results. Bansal, et al. [16] consider the parameters for cost and load balancing by Virtual Machine Tree (VMT) enhanced task scheduling algorithm and verified that the parameter for cost is not so effective with proposed algorithm. Razaque, et al. [17] put forward an efficient task scheduling algorithm that offers divisible task scheduling in view of network bandwidth and automatically implements the tasks when tasks are scheduled for the execution.

Most Efficient Server First (MESF) is a task scheduling scheme that schedules the tasks to maximize the energy aware servers of a data center. MESF decreases average task response time. Moreover, it also utilizes the equal amount of time and decreases the cost for the server expenses [18]. Thomas, et al. [19] propose a Min-min algorithm that takes into consideration both cloud users’ requirement and resource availability. Proposed algorithm decreases makespan of the tasks by analyzing task size. An Interaction Artificial Bee Colony (IABC) algorithm is presented for balancing of cloud loads, which improves assembly of the systems and schedules the tasks to VMs for its advance professional development [20]. For task scheduling, Raghavan, et al. [21] intend the meta-heuristic algorithms identified as BAT algorithm and Binary Bat Algorithm for the efficient workflow scheduling in cloud computing. To minimize the makespan of tasks scheduling in IaaS cloud, Abdulhamid, et al. [22] use a League Championship Algorithm (LCA) for the purpose of efficient tasks scheduling in IaaS cloud computing system.

Lin, et al. [23] design a non-linear programming method for determining the constrained multiport models problems, by bandwidth aware task scheduling (BATS), which is an innovative task scheduling algorithm. Furthermore, the algorithm allocates the appropriate amount of tasks to VMs, while including the CPU, energy, storage and network speed. Netjinda, et al. [24] emphasis on the situation that requires static task scheduling and consider that the workflows are intermittently implemented. To efficiently determine the optimal solutions, PSO algorithm needs to perform two important functions, exploitation and exploration. Wang, et al. [25] recommend the least Job time consuming algorithm and Load Balancing Genetic Algorithm (JLGA) to find the optimum task circulation categorization in a dynamic cloud environment. Furthermore, proposed algorithm decreases the makespan time for tasks by handling the workload of the complete system. Due to the VMs stack stays in a realistic condition, it keeps away from the unwanted sources and extra concerns. In addition, ACO-LB algorithm efficiently assembles the appropriate resources at job finishing point and assistances in resource allocation in a peer group [26]. Abdulhamid, et al, [27] propose Global LCA (GBLCA) algorithm for solving the non-deterministic problem of secure scheduling of tasks by minimizing the makespan and response time. Furthermore, Abdulhamid, et al, [28] use Dynamic Clustering LCA (DCLCA) algorithm for fault tolerance aware task scheduling by reducing the makespan and failure rate in cloud computing.

In cloud computing, features of task scheduling are discoursed by [29], which deliberates an algorithm for task scheduling that is designed based on genetic-ant colony algorithm. The benefit is having a resilient enthusiastic response of ant colony optimization (ACO) and compelling into interpretation the convergence ratio of the algorithm. Hung, et al. [30] propose a process for task scheduling, while keeping in view the clashes associated with expenses and network of cloud in order to reduce the recovery time for the enhancement and advancement of constancy. For the hybrid cloud, Wang, et al. [31] recommend the adaptive scheduling with QoS satisfaction algorithm, namely AsQ algorithm. It estimates finishing time and numerous optimization procedures to discover an adjacent optimum resource allocation strategy. Thus the utility ratio of the private cloud, the leasing expenditure and the completion time of tasks are enhanced.

An enhanced form of task scheduling in cloud computing is proposed by Zhao, et al [32], which takes the intelligence firefly algorithm into account. With the behavior of firefly algorithm, the cloud computing research demonstrates the extreme resolution for task scheduling. With the help of fuzzy clustering, Li, et al. [33] suggest an algorithm and model to distribute a suitable resources to tasks mapping. It achieves the desires of tasks and reserve for the influential resources. Wu, et al. [34] propose a task scheduling QoS driven algorithm based on MCT algorithm in cloud computing. Task Scheduling QoS (TS_QoS) algorithm computes the priority of task discussing to the appearances and at that point organizes the tasks with respect to their priority order. For optimizing task scheduling Gabi, et al. [35] propose Orthogonal Taguchi-based Cat Swarm Optimization (OTB-CSO) hybrid algorithm to minimize the delay in total task execution. The purpose is to reduce the makespan and degree of imbalance for all schedule tasks on VMs.

For reducing the imperfections of the cloud computing data center in resource management, to confirm that cloud computing provides superior QoS service. Ant colony optimization (ACO) is applied in the paradigm of cloud computing to manage the resource and schedule regarding to the actual QoS parameters required for the cloud computing [36]. A novel scheduling algorithm that proficiently schedules the computational tasks in a cloud computing and produces tree based data structure identified as a VMT. They transformed DFS, uses the suitable VMs for execution [37]. To attain a suitable task, advanced genetic algorithm is executed for resource scheduling in cloud computing. As a final point, experimental result based on cloudsim shows the accurateness of the scheduling algorithm with its strength [38].

An effective VMs allocation algorithm and job scheduling policies directly effect on the transaction between cloud providers and users. For this purpose, Cao, et al. [39] compare the various job scheduling policies including FCFS, SJNF, SJEF, LJNF and LJEF for resource utilization and cost optimization by using Python-based simulation package–SimPy. Further, He, et al [40] introduce and compare the five heuristic algorithms to evaluate the performance of CloudSim tool. Sequence Scheduling (SS), FCFS, Shortest Task First (STF), Balance Scheduling (BS) and Greedy Scheduling (GS) algorithms are used to solve the issue of task scheduling in cloud computing.

Patel, et al. [41] reviewed heuristic algorithms for the static task scheduling in cloud computing, consist of Opportunistic Load Balancing (OLB), MCT, MCT, Max-min, Min-min and Load Balancing Min-min (LBMM) and proposed Enhanced (LBMM) algorithm for static task scheduling in cloud computing. Moreover, detailed studies of several task scheduling algorithms are presented for the cloud computing by [42]. These algorithms are FCFS, RR, OLB, Min-min, Max-min, GA, SA, Switching Algorithm, Sufferage, etc. Also, a brief study of many scheduling parameters is discussed including the makespan, deadline, execution time, completion time, energy, performance, QoS and load balancing for task scheduling in cloud computing.

Akilandeswari and Srimathi [43] present the comparative analysis of static and dynamic task scheduling algorithms used by cloud providers in cloud computing. For static task scheduling FCFS, RR, Min-min and Max-min algorithms, while for the dynamic task scheduling ACO, GA, PSO and SA are proposed for implementation. Similarly, Thaman, et al. [44] present a taxonomy for task and job scheduling meta-heuristic and heuristic algorithms. These categorization are based on the goal and constraint oriented task scheduling algorithms. Tabak, et al. [45] present an algorithmic enhancement that asymptotically reduces the execution time of Min-min algorithm without affecting the quality of service. Further, the newly anticipated Min-min algorithm is combined with Max-min and Sufferage algorithm, to obtain two hybrid algorithms. The incentive of hybrid algorithms are discourse the disadvantage of Max-min in resolving problematic instances with highly skewed cost circulations and also improve the execution time results of Max-min algorithm.

## Rule based scheduling heuristics

In this section, the rule based scheduling heuristics algorithms are presented to lay down the foundation for task scheduling and we discussed their working in IaaS cloud computing system. In cloud computing, heuristic algorithms are designed to resolve the problematic issues faster than meta-heuristic algorithms, when their performance is too slow. Also, heuristic algorithms are used to find an optimum solution, when meta-heuristic algorithms failed to discover the precise or optimal solution. These are achieved by accuracy, completeness, optimal transaction or speed. It is considered to be a shortcut [46–48].

### First Come First Serve (FCFS)

FCFS algorithm is known to schedule and manage processes that automatically executes tasks or resource and precedes them by the order of their arrival demand of users. With FCFS algorithm, first arrival demand of task or resource is fulfilled first and then next demand in a queue will be executed once the one before it is complete. It is also based on the FIFO algorithm. It provides efficient, error-free and simple process for scheduling by saving the VMs or resources in cloud computing. CloudSim [12], [49], iFogSim [50] CEPSim [51] and GridSim [52] simulators used FCFS algorithm by default for the scheduling purpose of the tasks and resources in cloud and grid environment.

Abdulhamid, et al. [22] compare the LCA with three other existing algorithms including the FCFS, Best Effort First (BEF) and Last Job First (LJF) to estimate the performance of suggested LCA task scheduling algorithm by reducing the makespan time. Further, Jamali, et al. [53] contrast the PSO, GA and FCFS algorithms for minimizing the makespan, waiting time and enhancing the performance of given tasks sets. Lakra and Yadav [54] compare the multi-objective task scheduling algorithm with FCFS and priority scheduling algorithm to reduce the throughput for task scheduling. Moreover, Zuo, et al. [55] detail a multi objective ACO algorithm for enhancing the cost, makespan, resource utilization and time deadline for the task scheduling and compare the results with FCFS and Min-min algorithm. Raju, et al. [56] propose Deadline Aware Two Stage Scheduling and evaluate the metrics of average turnaround time, average waiting time and violation in deadlines to schedule VMs by comparing with FCFS and Shortest Job First (SJF) algorithm.

Li, et al. [57] compare the proposed Load Balancing Ant Colony Optimization (LBACO) algorithm with the basic Ant Colony Optimization (ACO) and FCFS for load balancing. Moreover, Mondal, et al. [58] compare the Stochastic Hill Climbing technique with FCFS and round robin (RR) for load balancing. Further, Dasgupta, et al. [59] compare the GA with FCFS, RR and Stochastic Hill Climbing algorithms for load balancing in cloud computing for task scheduling.

Sindhu and Mukherjee [60] present two algorithms, Longest Cloudlet Fastest Processing Element (LCFP) and Shortest Cloudlet Fastest Processing Element (SCFP) for scheduling tasks in a private cloud in order to attain the lowest makespan time. Also, FCFS is used in the study for the comparison of the performances of the algorithms in the simulation. Further, Sindhu and Mukherjee [61] present a bi-objective GA based on scheduler for resource scheduling that improves the makespan and resource utilization as evaluation with FCFS and RR algorithms. Similarly, Tawfeek, et al. [62] recommend a task scheduling policy based on ACO algorithm for optimal task scheduling and prove the enhanced makespan as comparison with FCFS and RR algorithms.

### Minimum Completion Time (MCT)

**Fig 2:**
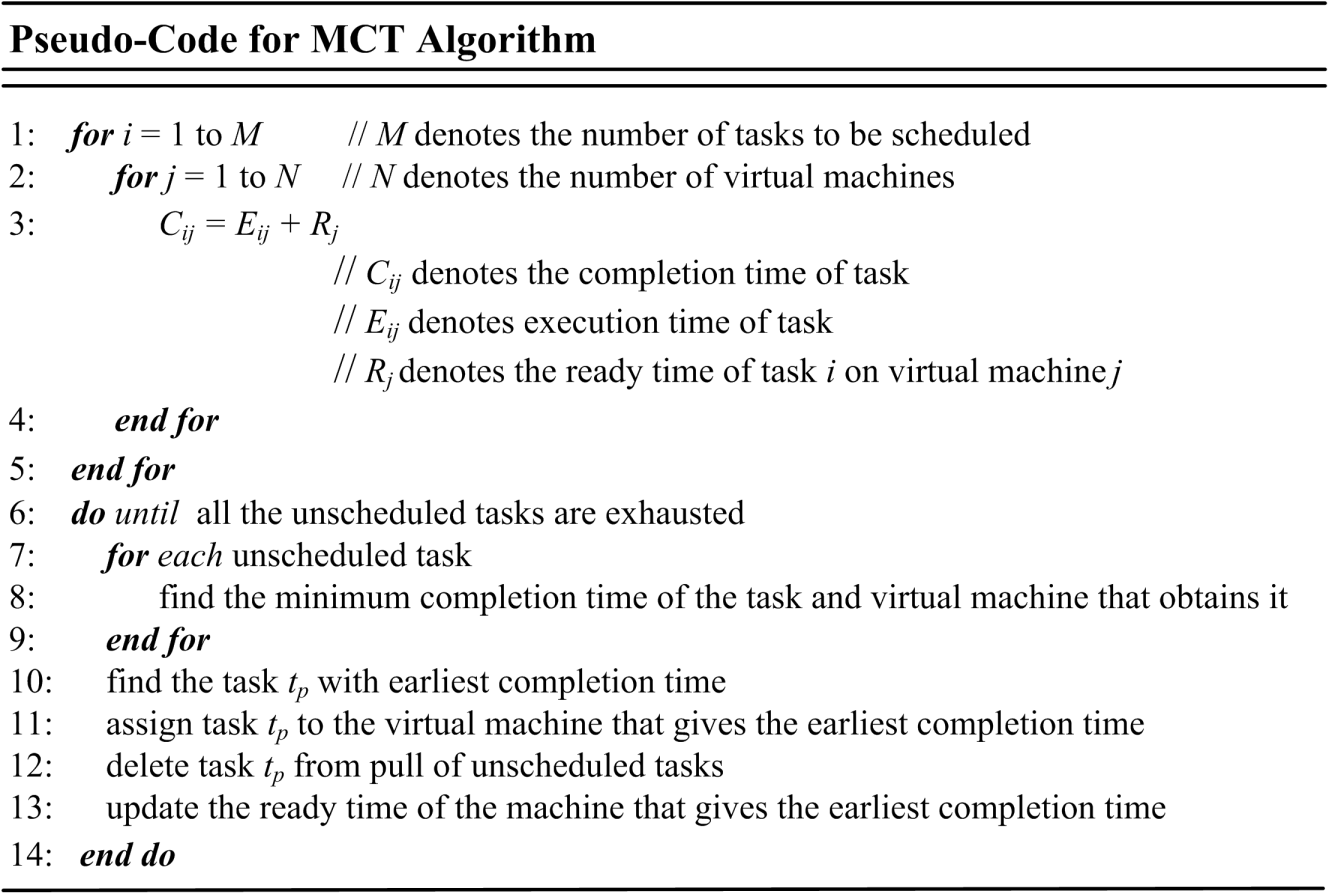
Pseudo-code of MCT algorithm.

Minimum Completion Time (MCT) algorithm assigns tasks to VMs or resources based on the best predictable completion time for that task in random order. Each task is assigned to the VM or resource that has earliest completion time. With MCT algorithm, some tasks are allocated to the VMs or resources having no minimum execution time. It tries to combine the advantages of OLB and MET algorithms while avoiding their drawbacks [63–66]. Fig 2 shows the pseudo-code for MCT algorithm.

Du Kim and Kim [67] recommend an innovative scheduling algorithm MECT consist of MET algorithm and MCT algorithm for on-line scheduling in heterogeneous computing systems. MECT shows better performance than the basic MET algorithm and MCT algorithm for reducing makespan.

### Minimum Execution Time (MET)

**Fig 3:**
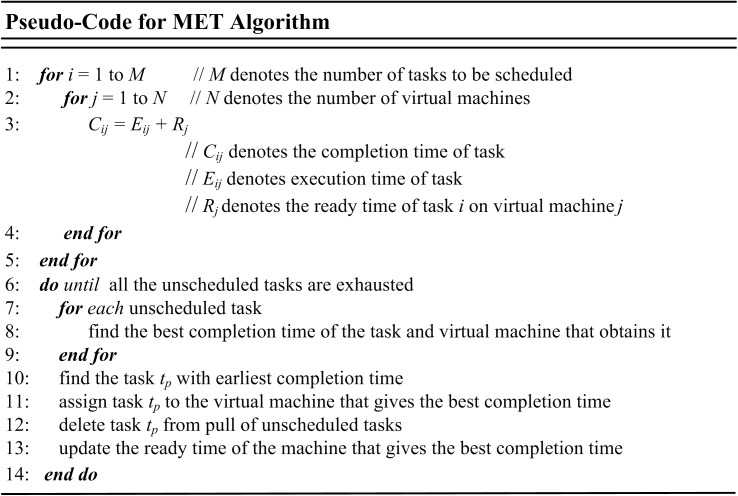
Pseudo-code of MET algorithm.

Minimum Execution Time (MET) algorithm assigns tasks to VMs or resources based on the best predictable completion time for that task without regard to resource availability. The core idea of MET is to assign a task to VM or resource based on minimum execution time, which sometimes result to high load imbalance since the assignment is not dependent on the availability [63–66]. Fig 3 shows the pseudo-code for MET algorithm.

### Max-min

**Fig 4:**
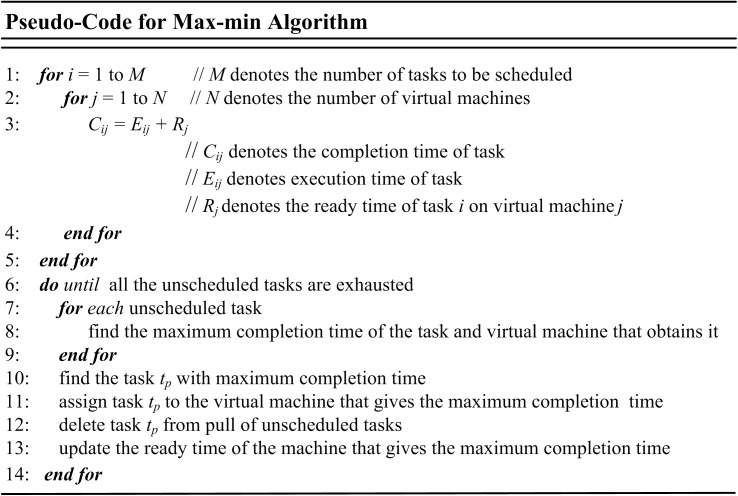
Pseudo-code of Max-min algorithm.

Similar to the Min-min algorithm, after determining the completion times for each task on all machines, the task with maximum completion time is scheduled on the consistent machine in the case of max-min and the process is repeated until all the tasks are scheduled [68]. In Min-min algorithm, the anticipation is that if more tasks are scheduled on machines that execute them earliest and fastest, smaller makespan will be obtained. Max-min algorithm is usually employed in a situation where there are fewer longer and shorter tasks. It can as well reduce starvation for the longer tasks since it will enable the longer tasks to be scheduled along with shorter ones. In this scenario, max-min guarantees better makespan and low degree of imbalance among machines [65, 69]. Fig 4 shows the pseudo-code for Max-min algorithm.

Mao, et al. [70] and Li, et al. [71] recommend Max-min algorithm for task scheduling to balance the load of elastic cloud. The recommended algorithm preserves a task position table to evaluate the real time workload of VMs and predictable execution time of tasks. The simulation outcomes express that Max-min algorithm increases the utilization of resource and reduces the response time for task scheduling.

The main objective of improved Max-min algorithm is assigned task with maximum execution time to the resource, which gives minimum completion time than basic Max-min algorithm. Improved Max-min algorithm is established on predictable execution time as a substitute of complete time, which gives lower makespan [72]. For task scheduling in cloud computing, performance of Max-min algorithm is not achieved the better results. To resolve this issue, Ming and Li [73] offer an enhanced algorithm MMST based on Max-min. It reduces the waiting time and improves the resource utilization of tasks. Also, MMST algorithm decreases the cost of cloud providers.

### Min-min

**Fig 5:**
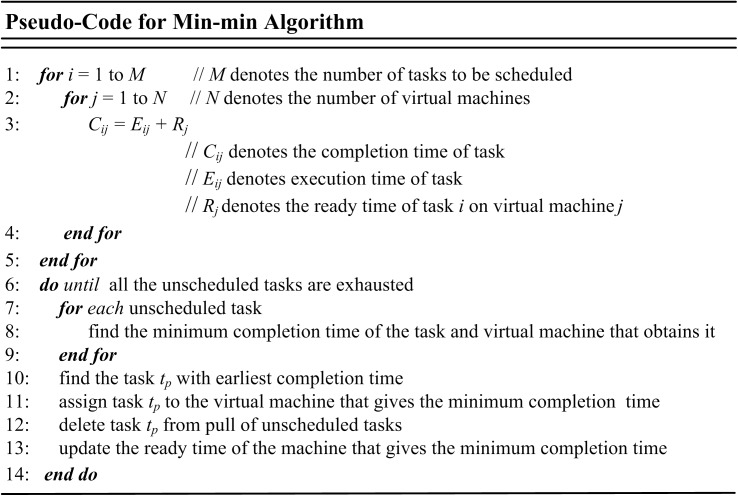
Pseudo-code of Min-min algorithm.

Min-min algorithm starts with a set of un-scheduled tasks and then determines the minimum completion times for each task on all machines. Then the task with generally minimum completion time is chosen and scheduled on the resultant machine [68]. The scheduled task is then detached from task list and the procedure is repeated until the all un-scheduled tasks are exhausted [65, 69, 74]. Fig 5 shows the pseudo-code for Min-min algorithm.

Wang and Yu [75] propose an improved Min-min algorithm for task scheduling for enhancing the proficiency of cloud computing system. However, Min-min algorithm continuously completes the minimum and entire execution time for task firstly, and then simply complete in the shortest period is characterized for scheduling. The consequences display that the algorithm is operative for the task scheduling in cloud computing. Further, Zhang and Xu [76] suggest a Min-min task scheduling algorithm based on QoS constraints in cloud computing. The suggested algorithm measures the similarity of resources or tasks, and then delivers to the users to fulfill their demands. Simulation results demonstrate that Mul-QoS-Min-Min performs better in enhancing the execution time and QOS satisfaction as compared with basic Min-min algorithm in cloud computing.

Tsai, et al [77] recommend a hybrid scheduling technique composed of Min-min and Longest Job First (LJF) to decrease the makespan for job scheduling in heterogeneous grid environment. Simulation results confirm that the performance of the suggested technique is better than others in reducing the makespan time. Patel, et al. [41] enhanced the Load Balancing Min-min (LBMM) algorithm for static task scheduling and maximize the utilization of resource in cloud computing. Further, Chen, et al. [78] introduce two novel algorithms for scheduling to enhance the makespan, resource utilization and user priority in cloud computing. LBIMM algorithm and PA-LBIMM algorithm are based on Min-min algorithm. The simulation results show that both the LBIMM and PALBIMM algorithms are outperformed than the basic Min-min algorithm to improve the completion time, load balancing and user priority.

### Sufferage

**Fig 6:**
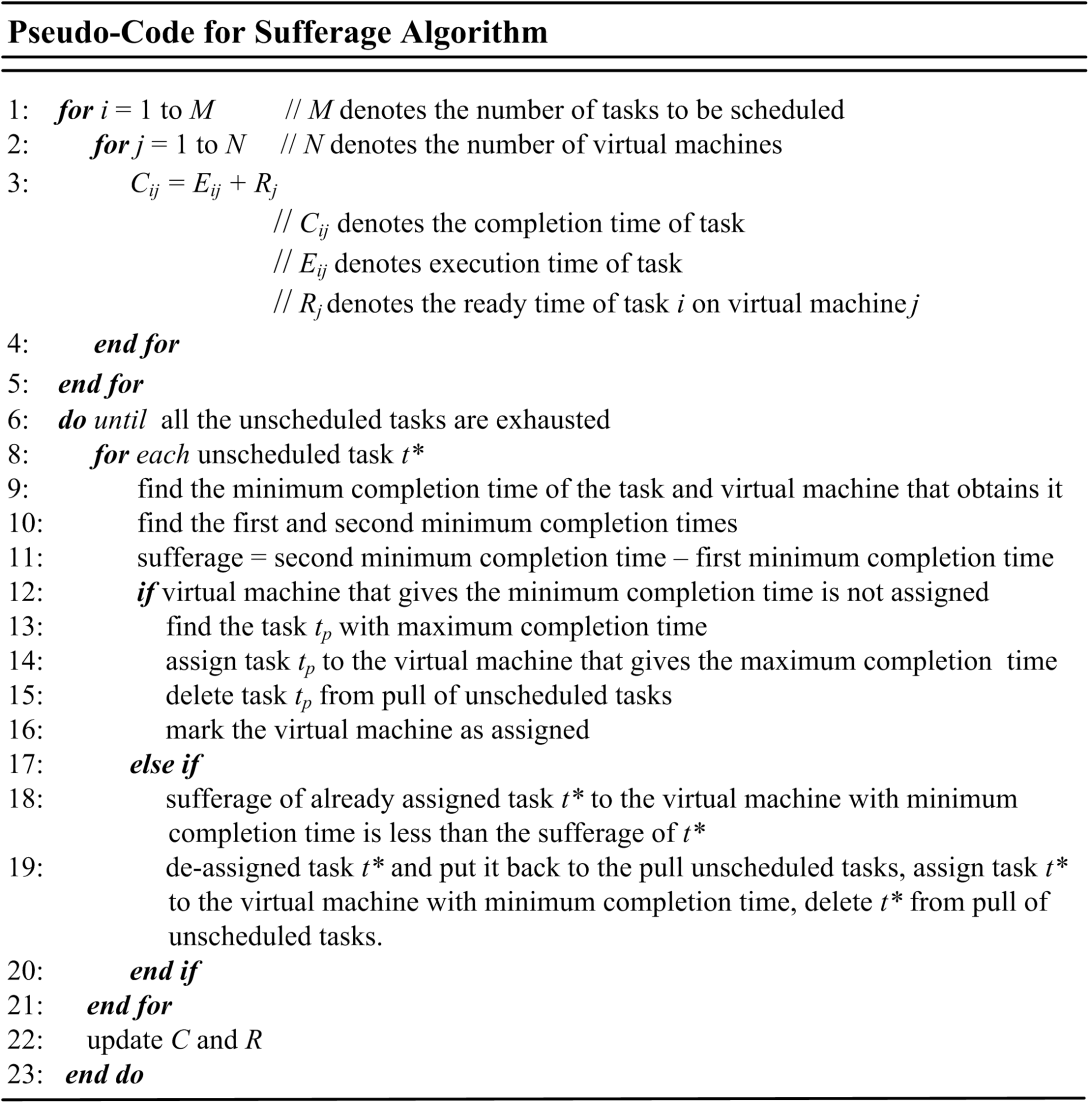
Pseudo-code of sufferage algorithm.

Sufferage algorithm starts by calculating values of tasks for the minimum and second minimum completion times. The differences of the values are determined in the second stage and task with a minimum difference (sufferage) is allocated to the consistent VM or resource. Then the task is detached from un-assigned task list and resource availability list is updated. The procedure is repeated until all the tasks are scheduled [66]. Fig 6 shows the pseudo-code for sufferage algorithm.

Han, et al. [79] propose a new scheduling algorithm composed of Sufferage algorithm and Min-min algorithm to improve the QoS for task scheduling. In the comparison of simulation results, proposed algorithm show better performance in decreasing the makespan for task scheduling for cloud computing.

## Performance metrics

This comparative analysis of performance metrics for task scheduling is based on cost, makespan, throughput and degree of imbalance. The performance metrics are discussed below:

### Cost

Cost means the total payment generate against the utilization or usage of resources, which is paid to the cloud providers by the cloud users. The main determination is to the growth of revenue and profit for cloud providers while reducing the expenses for cloud user with efficient utilization [80, 81]. Assume the cost of a VM varies from on another based on time substantial and VM’s specification as specified by the cloud providers, then Eq 1 holds for the cost of executing task of a VM.

$$Cost=\sum_{i=1}^{n}task^{i}\left(Ci\;*\;Ti\right)$$ (1)

where *Ci* represents the cost of *i*^*th*^ VM and *Ti* represents the execution time of *i*^*th*^ task.

### Degree of imbalance

Degree of imbalance (DI) describes the amount of load distribution amongst the VMs regarding to their execution competencies. Here, *T*_max_, *T*_min_ and *T*_a*vg*_ signify the maximum, minimum and average overall execution time of task among total VMs, correspondingly [14, 81].

$$DI=\frac{T_{\max}-T_{\min}}{T_{avg}}$$ (2)

### Makespan

Makespan is used to estimate the maximum completion time, by evaluating the finishing time of the latest task, when all tasks are scheduled. If the makespan of specific cloudlet or task is not minimized then the demand will not be completed on time [27, 82].

$$Makespan=max_{task^{i}}\left(Fnh_{Time}\right)$$ (3)

where *Fnh*_*Time*_ shows the finishing time of *i*^*th*^ task.

### Throughput

Throughput uses the consideration of total number of tasks, which are implemented successfully. In cloud computing, throughput means some tasks completed in a certain time period. Minimum throughput is required for task scheduling [81, 83].

$$Throughput=\sum_{task^{i}}\left(Exe_{Time}\right)$$ (4)

where *Exe*_*Time*_ shows the execution time of *i*^*th*^ task.

## Results and discussion

This section explains the simulation setup and results obtained after running the heuristic task scheduling algorithms. These algorithms are implemented in CloudSim simulator in homogeneous and heterogeneous environment with and without using workload traces. Cloud users, cloudlets, host, VMs and datacenter specification are presented in Tables 1 and 2 for homogeneous and heterogeneous environments. The larger cloudlets will enable the improvement perception in scalability the performance of the algorithms with the large problem sizes and fewer users’ demand. These algorithms are compared with each other on a set of parameters like cost, degree of Imbalance, makespan and throughput for task scheduling in IaaS cloud computing. For calculating the resource cost based on VM’s specification are considered, and Cost of resources as follow:$0.12, $0.13, $0.17, $0.48, $0.52 and $0.96 per hour [55, 84].

**Table 1:** Simulation parameters setting of CloudSim for homogeneous environment.

Sr. No	Entities	Parameters	Values
1	User	No of users	5
2	Cloudlet	No of cloudlets	100–1000
		Length	2000
3	Host	No of Host	2
		RAM	2048MB
		Storage	1000000
		Bandwidth	10000
4	Virtual Machine	No of VMs	15
		Type of Policy	Time Share
		RAM	512MB
		Bandwidth	1000
		MIPS	1000
		Size	10000
		VMM	Xen
		Operating System	Linux
		No of CPUs	2
5	Data Center	No of Data Centers	2

**Table 2:** Simulation parameters setting of CloudSim for heterogeneous environment.

Sr. No	Entities	Parameters	Values
1	User	No of users	10
2	Cloudlet	No of cloudlets	100–1000
		Length	2000
3	Host	No of Host	2
		RAM	20GB
		Storage	1TB
		Bandwidth	10GB
4	Virtual Machine	No of VMs	25
		Type of Policy	Time Share
		RAM	128 to 15360 MB
		Bandwidth	128 to 15360 MB
		MIPS	256 to 30720
		Size	10GB
		VMM	Xen
		Operating System	Linux
		No of CPUs	2
5	Data Center	No of Data Centers	2

### Homogeneous environment

In the homogeneous environment, we have fixed specification of the VMs to check the performances of the heuristic algorithms for task scheduling for IaaS cloud computing, while changing the number of cloudlet with and without the workload traces using HPC2N [85] in our simulation. Table 1 shows the setting of experimental parameters for CloudSim in homogeneous environment.

**Fig 7:**
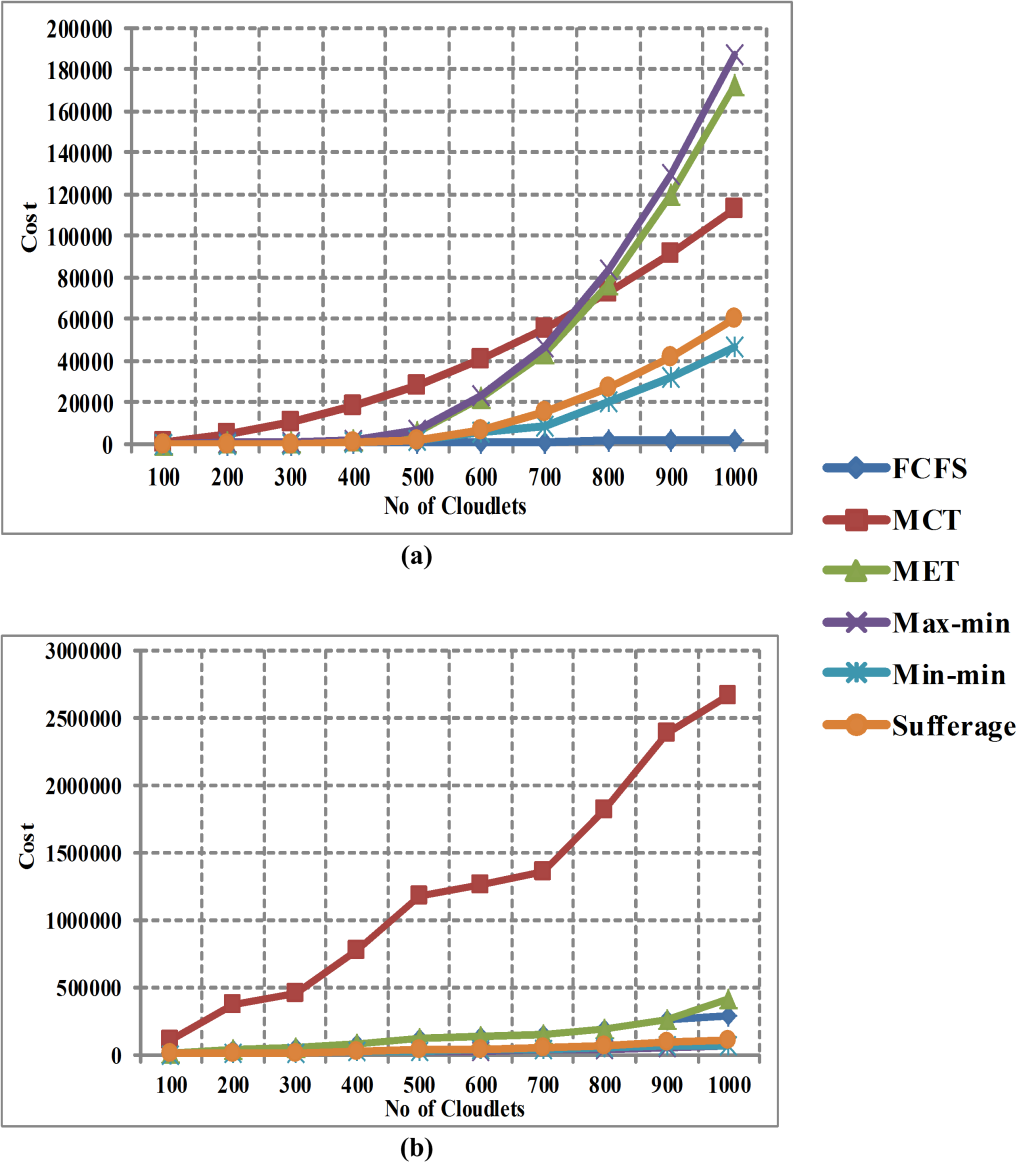
(a) Cost in Homogeneous Environment without Workload Traces and (b) Cost in homogeneous environment with workload traces.

Fig 7(A) shows the comparison of cost between FCFS, MCT, MET, Max-min, Min-min and Sufferage algorithms without using workload traces in homogeneous environment. The x-axis signifies number of cloudlets and y-axis signifies the cost per hour of the execution of tasks. The comparison outcomes show that the FCFS algorithm gives minimum cost than other heuristic algorithms without using the workload traces in homogeneous environment. Fig 7(B) shows the comparison of cost between FCFS, MCT, MET, Max-min, Min-min and Sufferage algorithms by using workload traces in homogeneous environment. The comparison outcomes demonstrate that the Max-min and Min-min algorithms give minimum cost (with minor difference) than other heuristic algorithms by using the workload traces in homogeneous environment.

**Fig 8:**
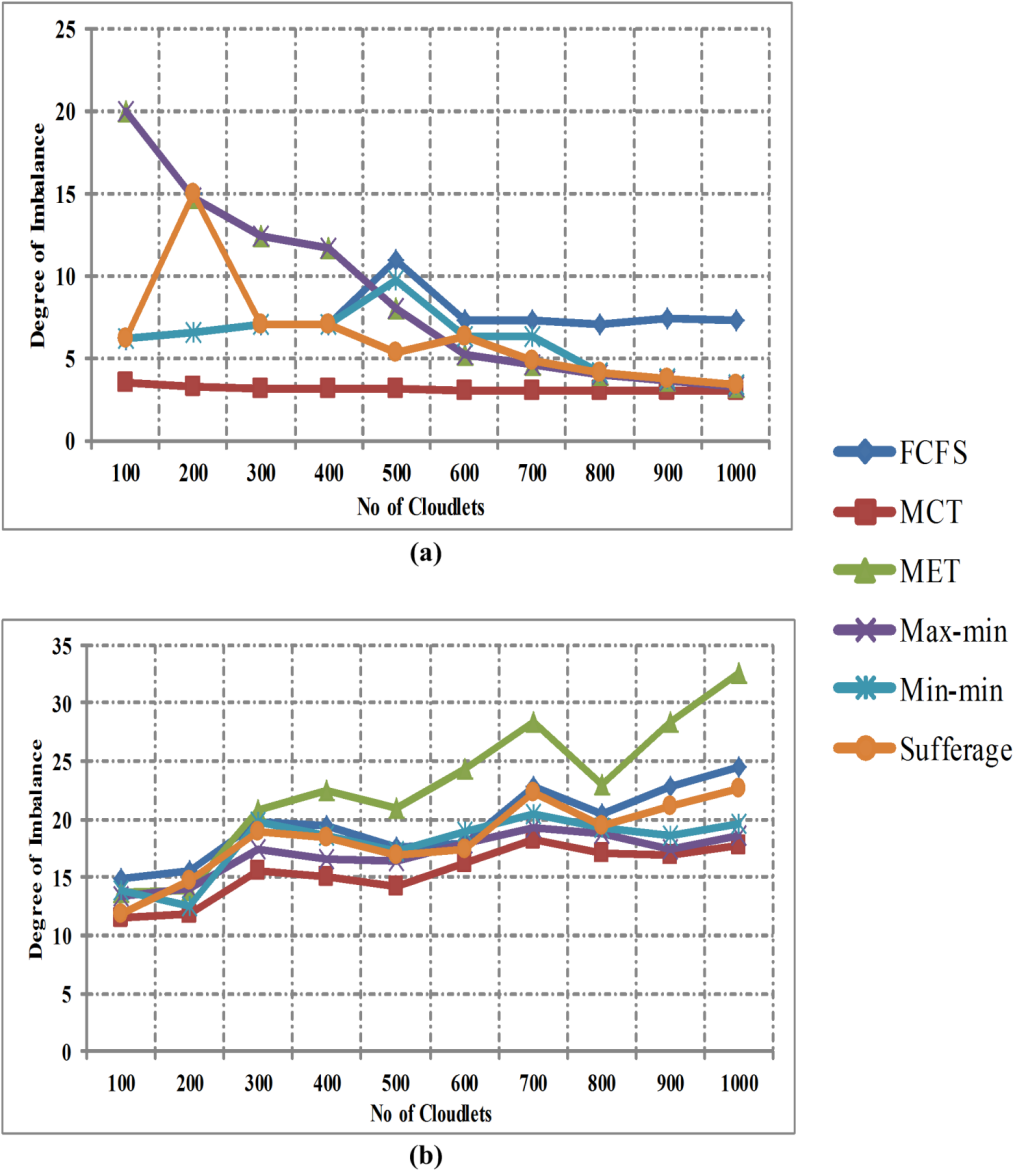
(a) Degree of Imbalance in homogeneous environment without workload traces and (b) Degree of Imbalance in homogeneous environment with workload traces.

Fig 8(A) shows the comparison of degree of imbalance between FCFS, MCT, MET, Max-min, Min-min and Sufferage algorithms without using workload traces and Fig 8(B) with workload traces in homogeneous environment. Horizontal line signifies number of cloudlets and vertical line signifies the degree of imbalance. The comparison results show that the MCT algorithm gives better degree of imbalance than other heuristic algorithms in both cases of homogeneous environment.

**Fig 9:**
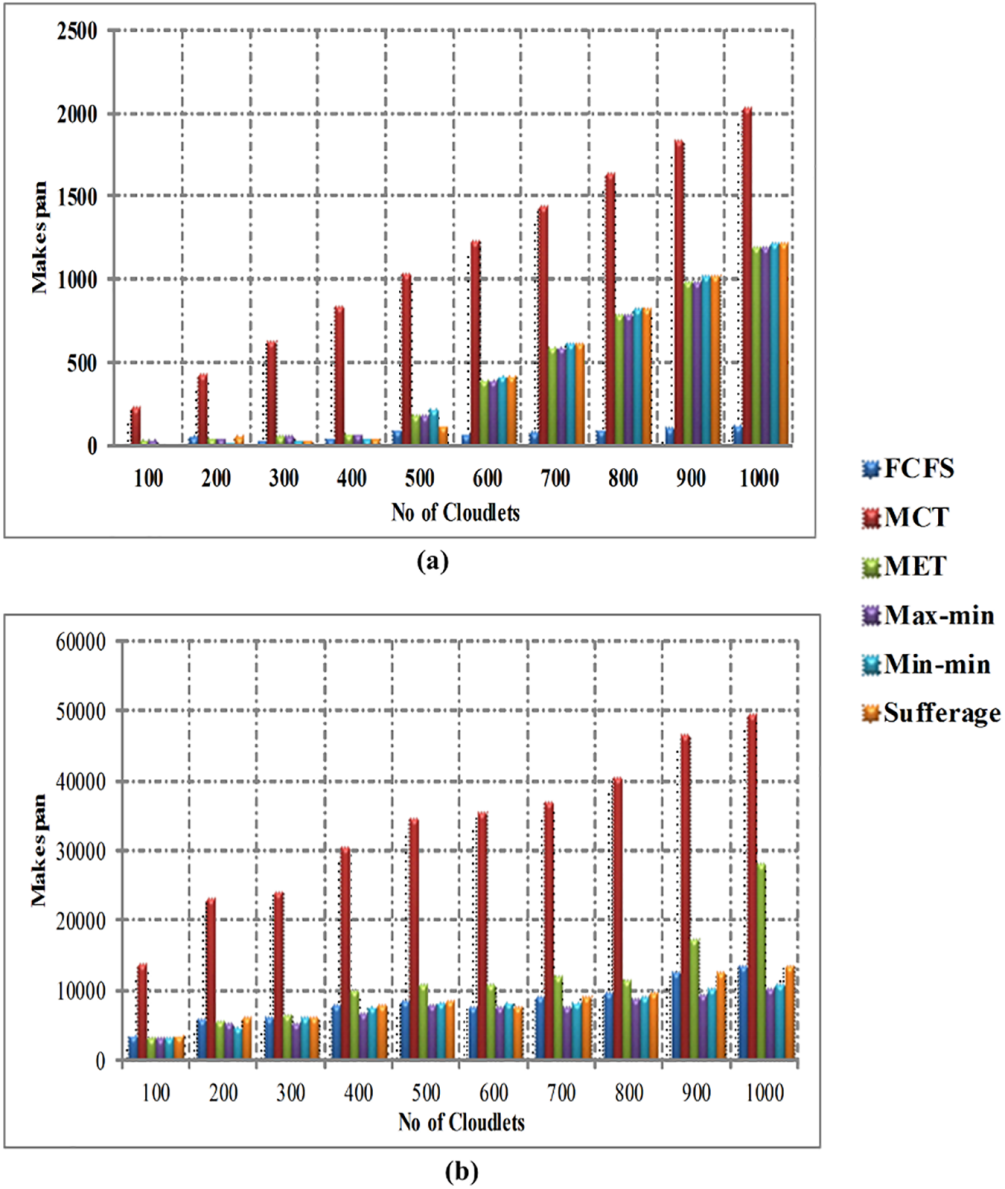
(a) Makespan time in homogeneous environment without workload traces and (b) Makespan time in homogeneous environment with workload traces.

In Fig 9(A), the comparison of makespan produced is shown between FCFS, MCT, MET, Max-min, Min-min and Sufferage algorithms without using workload traces in homogeneous environment. The x-axis indicates the number of cloudlets and the y-axis indicates the makespan time. When the numbers of cloudlets are less, then FCFS, Min-min and sufferage algorithms give enhanced makespan. When the number of cloudlets is increased, FCFS algorithm produces better makespan time in homogeneous environment without workload traces. Fig 9(B) illustrates the difference in makespan produced between FCFS, MCT, MET, Max-min, Min-min and Sufferage algorithms by using workload traces in homogeneous environment. The comparison of results clearly shows that the Max-min algorithm generates enhanced makespan than other heuristic algorithms by using the workload traces in homogeneous environment.

**Fig 10:**
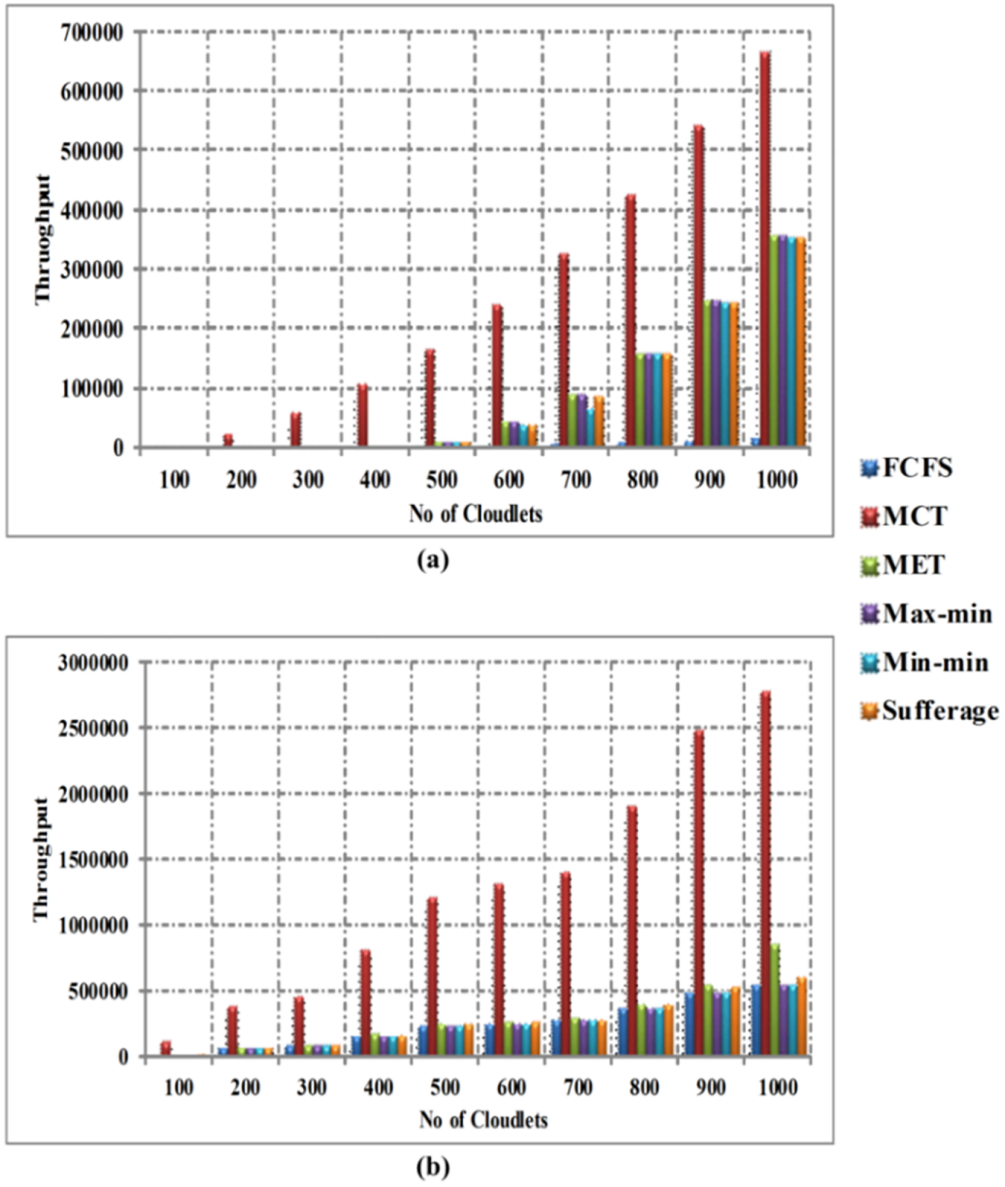
(a) Throughput time in homogeneous environment without workload traces and (b) Throughput time in homogeneous environment with workload traces.

Fig 10(A) explains the comparison of throughput between FCFS, MCT, MET, Max-min, Min-min and Sufferage algorithms without using workload traces in homogeneous environment. Horizontal axis denotes number of cloudlets and vertical axis denotes the throughput time. The simulation outcomes clearly prove that the Min-min algorithm provides better throughput than other heuristic algorithms, but the difference is not too much in homogeneous environment. Therefore, Sufferage and Max-min algorithms also show the better performance for throughput time. Fig 10(B) indicates the appraisal of throughput between FCFS, MCT, MET, Max-min, Min-min and Sufferage algorithms by using workload traces in homogeneous environment. The comparison of results clearly displays that the Max-min algorithm provides better throughput than other heuristic algorithms with using the workload traces, but difference is not too much than Min-min algorithm in homogeneous environment.

In homogenous environment, the specifications of all VMs are same as static. In this case, on the behavior of algorithms performances are depended. FCFS algorithm shows more efficient performance for the cost and makespan without workload traces. Similarly, MCT algorithm gives the better performance for measuring the degree of imbalance in both cases. However, Max-min and Min-min show good performance with workload traces for achieving the minimum cost, makespan and throughput.

### Heterogeneous environment

In the heterogeneous environment, the VMs are selected randomly with different RAM, Bandwith and MIPS, to check the performance of the heuristic algorithms for task scheduling for IaaS cloud computing [86].

Six different workload traces are used to evaluate the performance by cost, degree of imbalance, makespan and throughput in heterogeneous environment. Four of them are generated using the uniform, normal, left-skewed and right-skewed distribution presented as S01, S02, S03 and S04 respectively. Uniform distribution shows the equal amount of small, large and medium size tasks. Normal distribution represents on the more medium, while less small and large size tasks. Skewness is amount of asymmetric of probability distribution of tasks in the datasets. It can be left (negative) or right (positive). Left-skewed illustrates that the tail of the distribution is to the left of its mean, which includes the more small and less large size tasks the dataset. Hence the right-skewed denotes that the tail of the distribution is to the right of its mean which includes the less small and large size task in the data sets. These datasets show the behaviour of heuristics algorithm with different workloads.

S05 and S06 are generated from “Parallel Workload Archives” consist of HPC2N (High-Performance Computing Center North) [85] and NASA Ames iPCS/860 [87]. These workload archives are provided by “Ake Sandgren” and “Bill Nitzberg”, in the standard workload format (swf) recognized by the CloudSim tool. HPC2N contains the information of 527,371 tasks and NASA contains the information of 14,794 tasks. These workloads are mostly used to evaluate the performance of algorithms in cloud computing environment [14, 15, 88–91]. Table 2 shows the setting of experimental parameters for CloudSim in heterogeneous environment.

**Fig 11:**
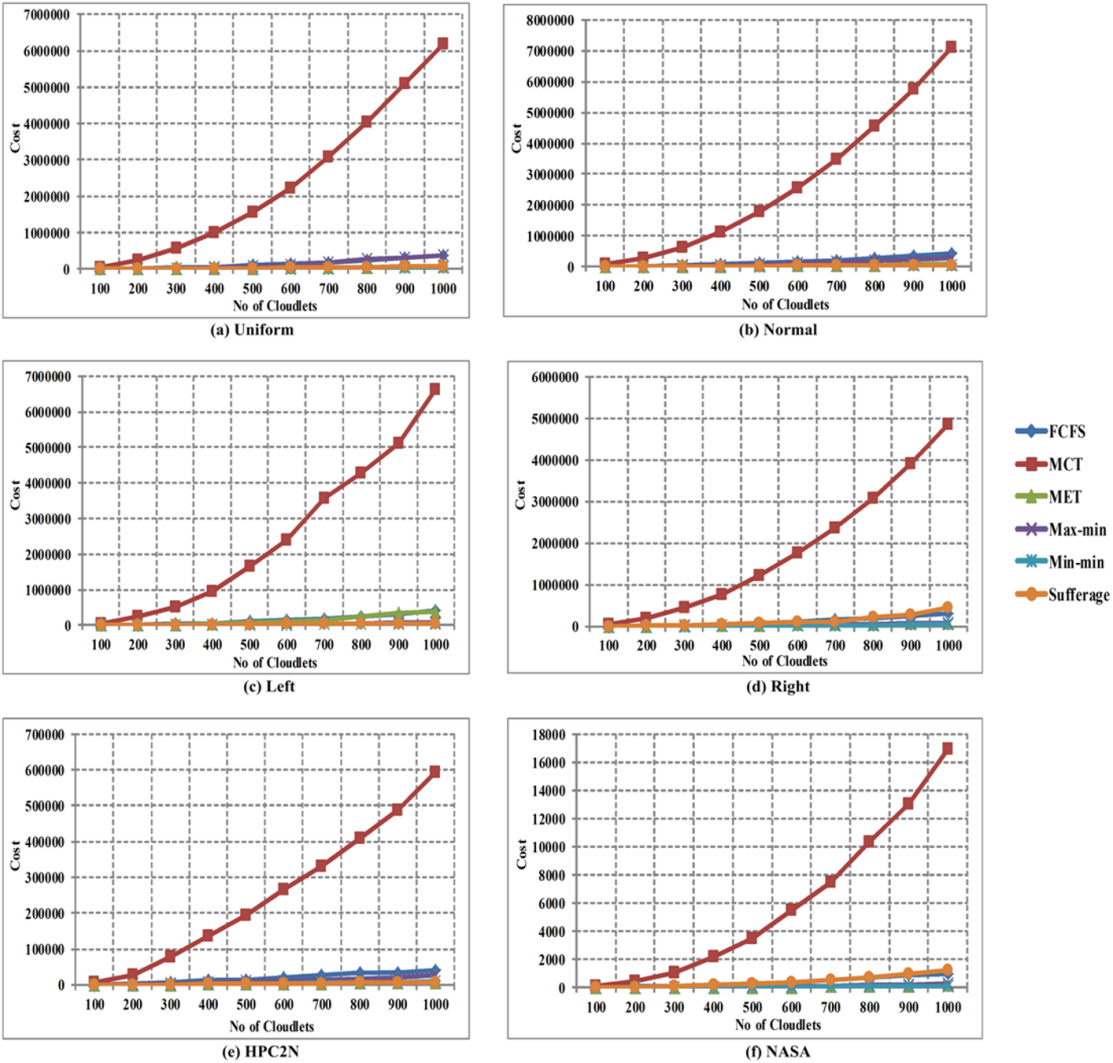
Cost in heterogeneous environment using (a) Uniform distribution, (b) Normal distribution, (c) Left-skewed, (d) Right-skewed (e) HPC2N and (f) NASA.

In Fig 11, the comparison of cost is shown between FCFS, MCT, MET, Max-min, Min-min and Sufferage algorithms with using workload traces including the Uniform, Normal, Left-Skewed, Right-Skewed, HPC2N and NASA in heterogeneous environment. The x-axis signifies the number of cloudlets and the y-axis signifies the cost per hour for the task execution. The comparison of results clearly expresses that the Min-min algorithm provides improved makespan than other heuristic algorithms in all six S01 to S06 for heterogeneous environment.

**Fig 12:**
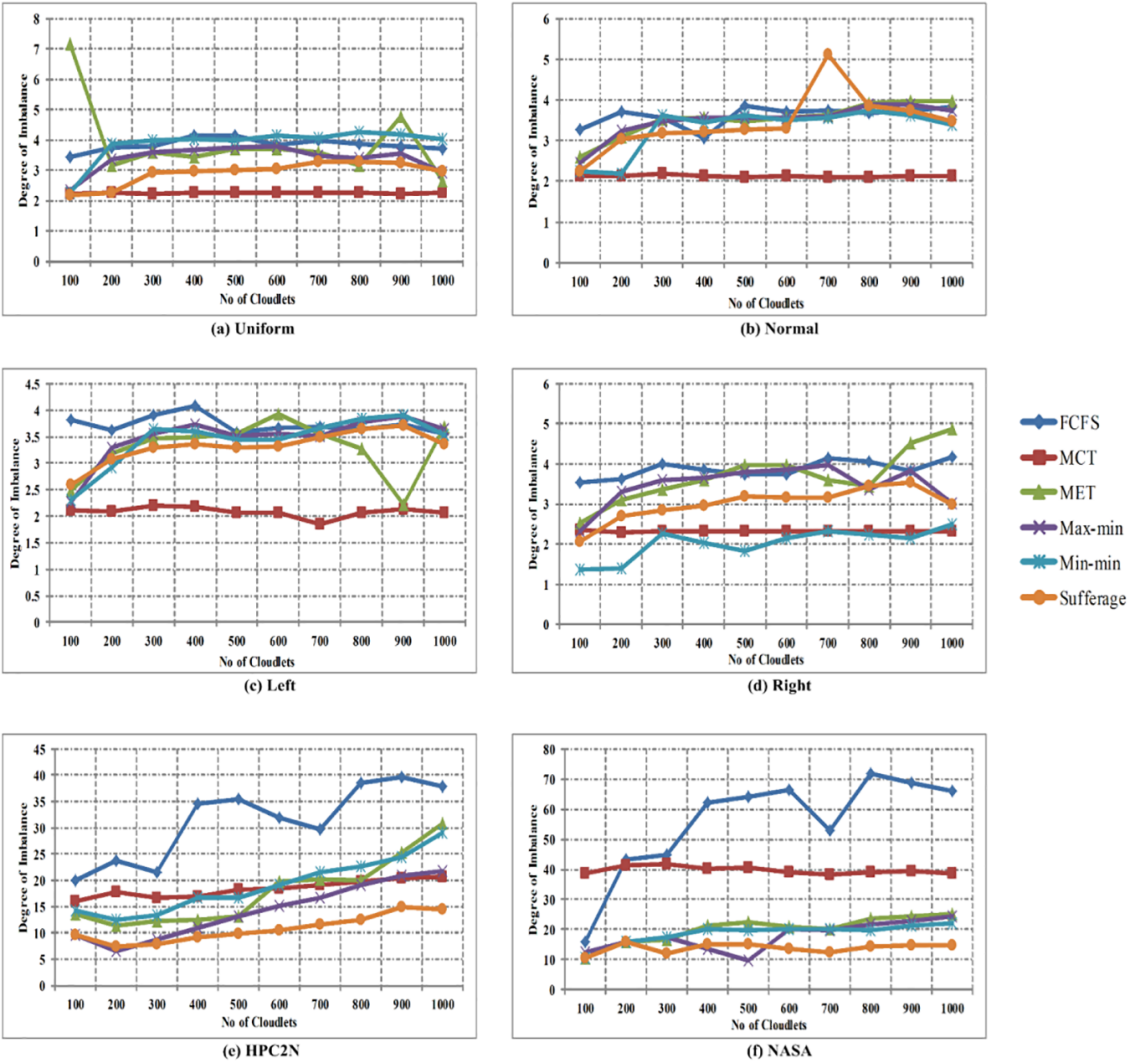
Degree of imbalance in heterogeneous environment using (a) Uniform distribution, (b) Normal distribution, (c) Left-skewed, (d) Right-skewed (e) HPC2N and (f) NASA.

In Fig 12, the comparison of degree of imbalance is shown between FCFS, MCT, MET, Max-min, Min-min and Sufferage algorithms with using workload traces including the Uniform, Normal, Left-Skewed, Right-Skewed, HPC2N and NASA in heterogeneous environment. The horizontal axis signifies the number of cloudlets and the vertical axis signifies the throughput time. The simulation results show that the MCT algorithm provides better performance in uniform distribution, normal distribution and left-skewed, FCFS in right-skewed and sufferage algorithm in HPC2N and NASA for homogeneous environment.

**Fig 13:**
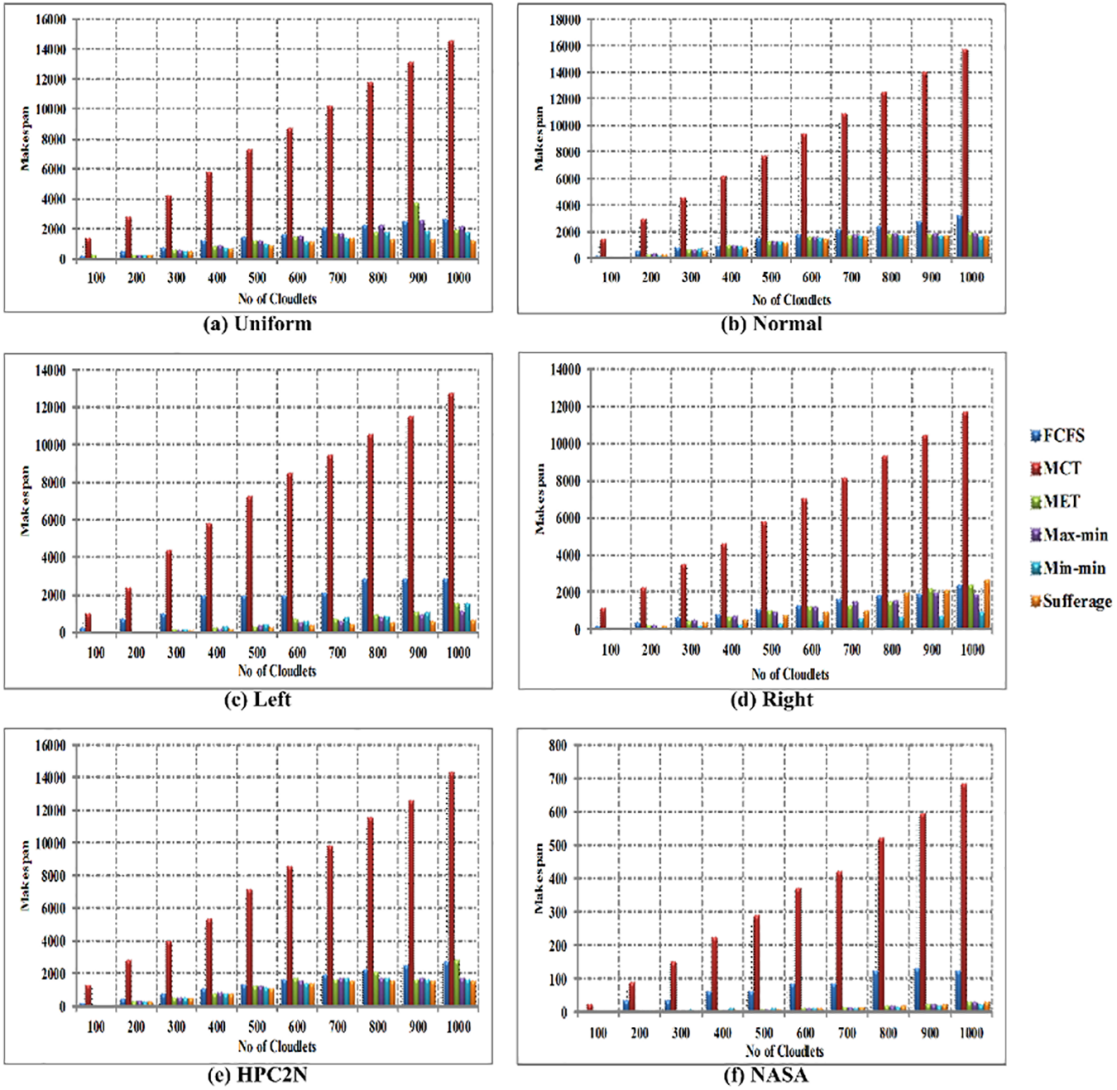
Makespan time in heterogeneous environment using (a) Uniform distribution, (b) Normal distribution, (c) Left-skewed, (d) Right-skewed (e) HPC2N and (f) NASA.

In Fig 13, the comparison of makespan time is shown between FCFS, MCT, MET, Max-min, Min-min and Sufferage algorithms with using workload traces including the Uniform, Normal, Left-Skewed, Right-Skewed, HPC2N and NASA in heterogeneous environment. The x-axis signifies the number of cloudlets and the y-axis signifies the makespan time. The comparison of results clearly shows that the Min-min algorithm provides improved makespan in uniform distribution, right-skewed and NASA, sufferage algorithm delivers in Left-skewed and HPC2N. While in normal distribution, both Min-min and sufferage algorithms give minimum makespan for heterogeneous environment.

**Fig 14:**
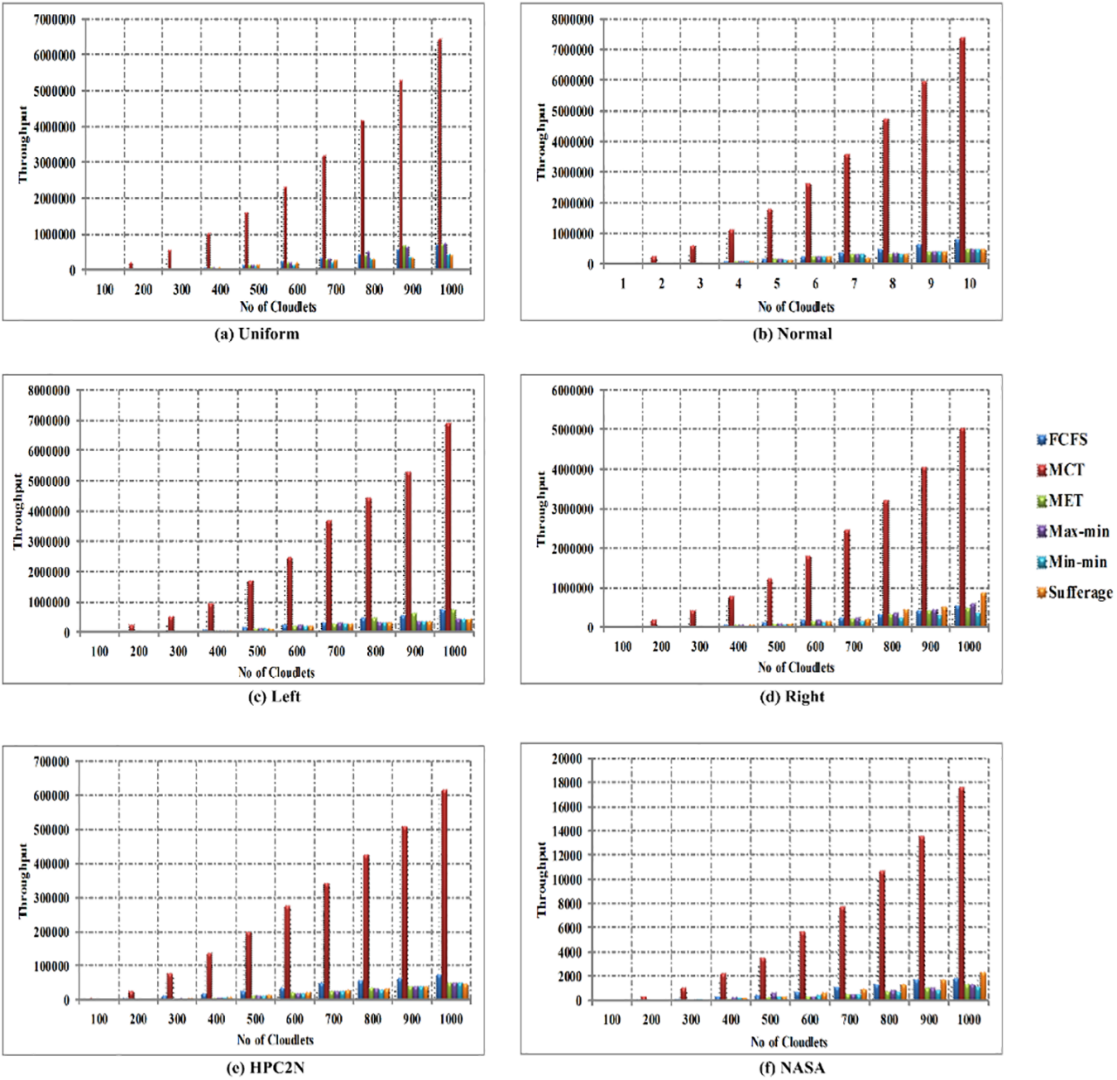
Throughput time in heterogeneous environment using (a) Uniform distribution, (b) Normal distribution, (c) Left-skewed, (d) Right-skewed (e) HPC2N and (f) NASA.

In Fig 14, the comparison of throughput time is shown between FCFS, MCT, MET, Max-min, Min-min and Sufferage algorithms with using workload traces including the Uniform, Normal, Left-Skewed, Right-Skewed, HPC2N and NASA in heterogeneous environment. Horizontal axis signifies the number of cloudlets and vertical axis signifies the throughput time. The comparison of simulation results clearly show that the Min-min algorithm offers enhanced throughput in S01, S02, S03, S04 and S06, while sufferage algorithm offer improved throughput in S05 for heterogeneous environment.

In the heterogeneous environment, Min-min algorithm outperformed in S01 to S06 for optimizing the cost, makespan and throughput. Although, the literatures of task scheduling show that Min-min is not used for optimizing the parameter of cost for task scheduling in cloud computing. Mostly it is applied for minimizing the makespan, throughput and degree of imbalance. So that researchers can use Min-min algorithm for the optimizing cost for task scheduling in cloud with improved version and hybrid with other heuristics and meta-heuristics algorithms.

Further, sufferage algorithm is also better performed for makespan and throughput in heterogeneous environment. FCFS and MCT show better results for achieving the degree of imbalance for task scheduling.

In both homogeneous and heterogeneous environment with and without workload traces, the performance of FCFS algorithm is not good in finding the optimal cost, makespan and throughput. Hence, FCFS algorithm has performed poorly in terms of the degree of imbalance for task scheduling in both situations in IaaS cloud computing. MET algorithm performs averagely in as compared with all selected heuristics algorithms in relations to cost, makespan and throughput, while in case of degree of imbalance its performance is not considered to be good in all setups of homogeneous and heterogeneous environment for task scheduling. MCT algorithm also performs poorly in finding the cost makespan and throughput time in both homogeneous and heterogeneous environment with and without workload traces, whereas it performs averagely as compared with other algorithms in finding the degree of imbalance in both scenarios for the task scheduling in IaaS cloud computing.

In case of Min-min algorithm, it shows average performance in finding makespan and throughput in homogeneous environment, while it gives and ideal results when finding optimum cost, makespan and throughput in heterogeneous environment with and without workload traces. Also, Min-min algorithm gives average results in searching the degree of imbalance in all four scenarios for the task scheduling in IaaS cloud computing. Max-min only archives the optimal makespan in homogeneous environment with workload traces, otherwise it shows average results as compared with all selected heuristic algorithms for task scheduling in finding the cost, makespan, throughput and degree of imbalance in IaaS cloud computing system. Sufferage algorithm always accomplishes the median results in searching for optimal the cost and degree of imbalance in both scenarios, but it shows the best results when trying to achieve an enhanced makespan and throughput in heterogeneous environment for task scheduling in IaaS cloud computing.

After evaluating the performances of heuristic algorithms, we conclude that Min-min is most suitable for optimizing the cost, makespan and throughput, while Max-min algorithm also shows good performance for achieving the optimal task scheduling in IaaS cloud computing. For the degree of imbalance, MET algorithm always shows better optimal results in attaining the optimal task scheduling in IaaS cloud computing. It is one the reasons that most researchers are using these heuristic algorithms as a standard for the comparison of their proposed techniques in cloud computing environment.

## Conclusion and recommendations

In conclusion, we present the performance comparison of heuristic algorithms for task scheduling in IaaS cloud computing system. These algorithms are executed with the help of CloudSim simulator in homogeneous and heterogeneous environments with and without using workload traces. These algorithms are compared with each other based on some parameters like cost, degree of imbalance, makespan and throughput. For the heuristics studied in this paper, overall Min-min algorithm performs better than other heuristics, while Max-min and sufferage algorithm give good results and MET algorithm always shows better performance in achieving the degree of imbalance for optimal task scheduling in IaaS cloud computing.

Further, we recommend that heuristic algorithm is adopted as a standard to compare new proposed algorithms to enhance and resolve task scheduling and other pressing research issues in IaaS cloud computing system. Due to the simplicity and easiness in implementation, heuristic algorithm shows faster and optimal outcomes. Hybridization of heuristic and meta-heuristic algorithms may give optimal results and cover the loopholes of each other to achieve the optimization of task scheduling in IaaS cloud computing. In future work, we want to compare the performance of meta-heuristic algorithms for task scheduling in IaaS cloud computing. Furthermore, we wish to improve the Min-min algorithm for optimizing the cost for task scheduling in cloud computing.
